# Sobolev type inequalities for compact metric graphs

**DOI:** 10.1186/s13660-018-1872-y

**Published:** 2018-10-05

**Authors:** Muhammad Usman

**Affiliations:** Department of Mathematics, Syed Babar Ali School of Sciences and Engineering, Lahore University of Management Sciences, Lahore, Pakistan

**Keywords:** 34L15, 81Q35, 35P15, 81Q10, Sobolev inequalities, Isoperimetric inequalities, Metric graphs, Quantum graphs, Laplace operator

## Abstract

In this paper analogues of Sobolev inequalities for compact and connected metric graphs are derived. As a consequence of these inequalities, a lower bound, commonly known as Cheeger inequality, on the first non-zero eigenvalue of the Laplace operator with standard vertex conditions is recovered.

## Introduction

For a compactly supported smooth function *h* on $\mathbb{R}^{n}$ the classical Sobolev inequality [30] and Gagliardo–Nirenberg inequality [14, 26] state the existence of positive constants *C* and *C̃* such that

1 $$ \int_{\mathbb{R}^{n}} \vert \nabla h \vert \,dx \geq C(n) \biggl( \int_{\mathbb{R}^{n}} \vert h \vert ^{\frac{n}{n-1}} \,dx \biggr)^{\frac{n-1}{n}} ,\quad n>1 $$

and

2 $$ \biggl( \int_{\mathbb{R}^{n}} \vert \nabla h \vert ^{2} \,dx \biggr)^{\frac{1}{2}}\geq \widetilde{C}(n) \biggl( \int_{\mathbb{R}^{n}} \vert h \vert ^{\frac{2n}{n-2}} \,dx \biggr)^{\frac{n-2}{2n}} ,\quad n>2 $$

hold. Inequalities (1) and (2) follow from a more general Gagliardo–Nirenberg–Sobolev (GNS) inequality,

3 $$ \biggl( \int_{\mathbb{R}^{n}} \vert \nabla h \vert ^{p}\,dx \biggr)^{\frac{1}{p}}\geq C(n,p) \biggl( \int_{\mathbb{R}^{n}} \vert h \vert ^{\frac{np}{n-p}} \,dx \biggr)^{\frac{n-p}{np}}, $$

where $1\leq p < n$. Inequalities (1) and (2) are obtained by choosing $p=1$ and $p=2$, respectively, in (3). These inequalities are important in the study of PDEs, heat kernel and spectral estimates (see, e.g., [10, 11]).

On the other hand, the corresponding versions of these inequalities for discrete graphs were obtained by Chung and Yau (see [8, 9]). The discrete analogue of (1) and (2) depend on a parameter associated with a graph, which we call the isoperimetric dimension of the graph, and a constant, which we call the isoperimetric constant of the graph. We define them as follows:

Let *G* denote a graph with vertex set $V(G)$. The graph *G* has an *isoperimetric constant* $C_{\delta}$ depending on the *isoperimetric dimension* *δ* if the number of edges between every subset *Z* of the vertex set $V(G)$ and its compliment *Z̄*, denoted by $|E(Z, \bar{Z})|$, satisfies

$$ \bigl\vert E(Z, \bar{Z}) \bigr\vert \geq C_{\delta}\bigl( \operatorname{Vol}(Z)\bigr)^{\frac{{\delta -1}}{{\delta}}}, \quad \mbox{whenever } \operatorname{Vol}(Z)\leq \operatorname{Vol}(\bar{Z}). $$

Here $\operatorname{Vol}(Z)$ denotes the sum of the valencies of all vertices in *Z*. Let $h: V(G)\rightarrow\mathbb{R}$ be an arbitrary function and $\tau_{h}$ is the largest value such that

$$\sum_{h(v)< \tau_{h}}d_{v}\leq\sum _{h(u)\geq\tau_{h}} d_{u} $$

with $d_{u}$ being the valency of the vertex *u*.

For a connected graph *G*, the discrete analogue of the Sobolev inequalities state the existence of positive constants $C_{1}=\frac {C_{\delta}(\delta-1)}{\delta}$ and $C_{2}=\frac{\delta-1}{2\delta C_{\delta}^{-1/2}}$ such that

4 $$ \sum_{u\sim v} \bigl\vert h(u)-h(v) \bigr\vert \geq C_{1} \biggl(\sum_{v} \bigl\vert h(v)-\tau_{h} \bigr\vert ^{\frac{\delta }{\delta-1}} d_{v} \biggr)^{\frac{\delta-1}{\delta}}, \quad\delta>1 $$

and

5 $$ \biggl(\sum_{u\sim v} \bigl\vert h(u)-h(v) \bigr\vert ^{2} \biggr)^{\frac{1}{2}}\geq C_{2} \min_{\tau _{h}} \biggl(\sum _{v} \bigl\vert h(v)-\tau_{h} \bigr\vert ^{\eta}d_{v} \biggr)^{\frac{1}{\eta}}, \quad\delta>2 $$

holds. Here $\eta=\frac{2\delta}{\delta-2}$ and $u\sim v$ means *u* and *v* are neighbors.

Inequalities such as (4) and (5) play an important role in the study of heat kernel and spectral estimates of discrete Laplacian on graphs. For instance, the lower bound on the *k*th eigenvalue $\lambda_{k}$ of the discrete Laplacian on a connected graph *G* is obtained by using inequality (5) as

$$\lambda_{k} \geq C_{3} \biggl(\frac{k}{\operatorname{Vol}(G)} \biggr)^{\frac{2}{\delta}}, $$

where the constant $C_{3}$ depends on *δ*.

One can consider a generalization of discrete graphs by identifying each edge by an interval of the real line instead of an ordered pair of vertices. In this way, one can define a distance function on such graphs, which can be the smallest path length between two points on the graph. This new object, with a metric defined on it, is called a *metric graph*. In addition, we can define ordinary differential operators on each edge with certain boundary conditions at the vertices. Boundary conditions or vertex conditions are chosen in a way which makes the overall operator self-adjoint on the graph.

Let Γ denote a metric graph with *N* being the number of edges and denote the *j*th edge by $e_{j}$ which is identified by an interval of the real line, i.e., $e_{j}=(x_{2j-1}, x_{2j})$, $j=1,2,\dots, N$. In the Hilbert space $L_{2}(\Gamma):=\bigoplus\sum _{j=1}^{N} L_{2}(e_{j})$, the Laplace operator (6) with standard conditions (7), which are also known as Kirchhoff, Neumann or free conditions, is a self-adjoint operator

6 $$\begin{aligned}& H h:= -h'' , \end{aligned}$$

7 $$\begin{aligned}& \textstyle\begin{cases} h \mbox{ is continuous at the vertex V},\\ \sum _{x_{j}\in V} \partial h(x_{j})=0. \end{cases}\displaystyle \end{aligned}$$

The extended normal derivative is $\partial h(x_{j}):=\lim_{x\rightarrow x_{j}}\frac{d}{dx}h(x)$ if $x_{j}$ is the left-end point and $\partial h(x_{j}):=\lim_{x\rightarrow x_{j}}-\frac{d}{dx}h(x)$ if $x_{j}$ is the right-end point. For the description of all possible vertex conditions for which (6) is self-adjoint, see [16, 18]. The pair of a metric graph and a self-adjoint differential operator is called a *quantum graph*. Such objects naturally arise in different areas of mathematics, science and engineering when analyzing various processes in systems which, locally, look like a thin neighborhood of a graph. In the last two decades, quantum graphs have evolved as an interesting branch of mathematical physics and have found many useful applications in physics, particularly in quantum chaos [15, 19] and mesoscopic physics [12, 13, 18]. For a detailed study of quantum graphs, we refer the reader to [3, 20–22, 29].

Although a quantum graph is fundamentally a different object from a discrete graph, in some special situations their spectra are related to each other. For example, it is well known (see [2, 4–6, 27, 31]) that if all edges of a compact metric graph are of the same length then the set of eigenvalues $\{ \lambda_{j}: \lambda_{j}/\pi^{2} \notin\mathbb{Z}\}$ of the Laplace operator (6) with vertex conditions (7) is related to the set of eigenvalues $\mu_{j}$ of the normalized discrete Laplacian as

$$1-\cos (\sqrt{\lambda_{j}} )=\mu_{j},\quad \mu_{j} \neq0,2. $$

Therefore, it is a natural question to ask whether it is possible to derive some functional inequalities of the type discussed above for metric graphs and whether one can obtain some estimates on the spectrum of the related quantum graphs? In this paper, we try to answer this question by deriving analogues of (3) for compact and connected metric graphs. A different version of GNS inequalities for non-compact metric graphs has been used by Adami, Serra and Tilli [1] to study ground states of certain NLSE.

The plan of the paper is as follows. In the next section we fix the notation and state our main results, Theorems 2.1 and 2.3. Section 3 contains proofs of the main results. Functional inequalities involving the graph’s Cheeger constant is the theme of Sect. 4. As a consequence of Theorem 4.3, we recover the well known Cheeger inequality for quantum graphs, which gives a lower bound on the lowest non-zero eigenvalue of the Laplacian (6) with standard conditions (7) on the vertices. For quantum graphs the same lower bound, Corollary 4.4, was first obtained by Nicaise [25, Theorem 3.2] and also by Post [28, Theorem 6.1].

## Main results

Let Γ be a compact and connected metric graph. We say that Γ has an *isoperimetric constant* $C_{\gamma}$ depending on the *isoperimetric dimension* *γ* if for every subgraph *Z* of Γ, the number of edges that depart from *Z*, denoted by $|\partial Z|$, satisfies

8 $$ \vert \partial Z \vert \geq C_{\gamma}\bigl( \operatorname{Vol}(Z)\bigr)^{\frac{{\gamma -1}}{{\gamma}}}, \quad \mbox{whenever } \operatorname{Vol}(Z)\leq \operatorname{Vol}(\bar{Z}). $$

Here *Z̄* denotes the complement of *Z*. For a non-negative function *h* on the metric graph Γ, we define

9 $$ Z^{+}_{h}(t):=\bigl\{ x \in\Gamma: h(x)>t\bigr\} $$

and

10 $$ Z^{-}_{h}(t):=\bigl\{ x \in\Gamma: h(x) \leq t\bigr\} . $$

Here $t\geq0$. It is easy to observe that there always exists a non-negative number $t_{h}$ such that $\operatorname {Vol}(Z^{+}_{h}(t_{h}))=\operatorname{Vol}(Z^{-}_{h}(t_{h}))$ and therefore $\operatorname{Vol}(Z^{+}_{h}(t))\leq\operatorname{Vol}(Z^{-}_{h}(t))$ for all $t\geq t_{h}$. In addition, if $h=c\geq0$ is a constant function then we set $t_{h}=c$.

Let **V** denote the set of vertices of Γ. We say that $h\in C^{1}(\Gamma)$ if $h|_{e_{j}}\in C^{1}(e_{j})$ and *h* satisfies the standard matching conditions (7) at all the vertices. The following two theorems are our main results:

### Theorem 2.1

*For* $h \in C^{1}(\Gamma)$, $h \geq0$ *and for* $\gamma>1$, *the following inequality holds*:

11 $$ \int_{\Gamma}\bigl\vert h'(x) \bigr\vert \,dx \geq C_{\gamma}\biggl( \int_{\Gamma}\bigl\vert h(x)-t_{h} \bigr\vert ^{\frac{\gamma}{\gamma-1}} \,dx \biggr)^{\frac{\gamma-1}{\gamma}}. $$

### Corollary 2.2

*Let* *h* *satisfy the conditions of the above theorem*. *Then*, *for* $\gamma >1$, *we have the following inequality*:

12 $$ \int_{\Gamma}\bigl\vert h'(x) \bigr\vert \,dx \geq C_{\gamma}2^{-1/\gamma} \biggl( \int_{\Gamma}\bigl\vert h(x) \bigr\vert ^{\frac{\gamma}{\gamma-1}} \,dx \biggr)^{\frac{\gamma-1}{\gamma }}-C_{\gamma}t_{h} \bigl(\operatorname{Vol} \bigl(Z^{+}_{h}(0)\bigr) \bigr)^{\frac{\gamma -1}{\gamma}}, $$

*where* $Z^{+}_{h}(0)=\{x\in\Gamma: h(x)>0\}$.

### Theorem 2.3

*For* $h \in C^{1}(\Gamma)$, $h \geq0$ *and for* $\gamma>1$ *and integer* $q\geq2$, *the following inequality holds*:

13 $$\begin{aligned} \biggl( \int_{\Gamma}\bigl\vert h'(x) \bigr\vert ^{\frac{q \gamma}{q + \gamma-1}} \,dx \biggr)^{\frac{q+\gamma-1}{q \gamma}} & \geq C_{\gamma,q} \biggl( \int_{\Gamma}\bigl\vert h(x) \bigr\vert ^{\frac{q \gamma}{\gamma-1}} \,dx \biggr)^{\frac{\gamma-1}{q\gamma }} \\ & \quad{}-C_{\gamma,q}\Theta(h,\gamma,q,\Gamma) \biggl( \int_{\Gamma}\bigl\vert h(x) \bigr\vert ^{\frac{q \gamma}{\gamma-1}} \,dx \biggr)^{\frac{(\gamma -1)(1-q)}{q\gamma}}, \end{aligned}$$

*where* $C_{\gamma,q}=\frac{2^{-1/\gamma}C_{\gamma}}{q}$ *and* $\Theta (h,\gamma,q,\Gamma)=2^{1/\gamma} t_{h^{q}}\operatorname {Vol}(Z^{+}_{h^{q}}(0))^{\frac{\gamma-1}{\gamma}}$.

### Remark 2.4

If $t_{h}=0$, then inequality (11) reduces to

$$\int_{\Gamma}\bigl\vert h'(x) \bigr\vert \,dx \geq C_{\gamma}\biggl( \int_{\Gamma}\bigl\vert h(x) \bigr\vert ^{\frac {\gamma}{\gamma-1}} \,dx \biggr)^{\frac{\gamma-1}{\gamma}}, $$

and if $t_{h^{q}}=0$, then inequality (13) becomes

$$\biggl( \int_{\Gamma}\bigl\vert h'(x) \bigr\vert ^{\frac{q \gamma}{q + \gamma-1}} \,dx \biggr)^{\frac{q+\gamma-1}{q \gamma}} \geq C_{\gamma,q} \biggl( \int_{\Gamma}\bigl\vert h(x) \bigr\vert ^{\frac{q \gamma}{\gamma-1}} \,dx \biggr)^{\frac{\gamma-1}{q\gamma}}. $$

## Proofs of the main results

In this section we prove our main results. We closely follow the argument of Cheeger [7] and Maz’ya [23, 24]. A similar argument was used in the case of discrete graphs [8, 9]. We will need the following lemma.

### Lemma 3.1

*For* $\gamma> 1$, *the following inequalities hold*:

14 $$\begin{aligned}& \int_{t_{0}}^{\infty} \operatorname{Vol} \bigl(Z^{+}_{h}(t)\bigr)^{\frac{\gamma -1}{\gamma}}\,dt \geq \biggl( \int_{\Gamma}\bigl(h(x)-t_{h}\bigr)_{+}^{\frac{\gamma }{\gamma-1}}\,dx \biggr)^{\frac{\gamma-1}{\gamma}}, \end{aligned}$$

15 $$\begin{aligned}& \int_{0}^{t_{0}} \operatorname{Vol} \bigl(Z^{-}_{h}(t)\bigr)^{\frac{\gamma-1}{\gamma }}\,dt \geq \biggl( \int_{\Gamma}\bigl(h(x)-t_{h}\bigr)_{-}^{\frac{\gamma}{\gamma -1}}\,dx \biggr)^{\frac{\gamma-1}{\gamma}}. \end{aligned}$$

### Proof

For simplicity we put $p=\frac{\gamma}{\gamma-1}$ and, using the definition of Lebesgue integral, we write

16 $$ \begin{aligned}[b] \biggl( \int_{\Gamma}\bigl(h(x)-t_{h} \bigr)_{+}^{p} \,dx \biggr)^{\frac{1}{p}} & = \biggl( \int_{0}^{\infty}\operatorname{Vol}\bigl(Z^{+}_{h}(t) \bigr) d(t-t_{h})_{+}^{p} \biggr)^{\frac{1}{p}} \\ & = \biggl( \int_{t_{h}}^{\infty}\operatorname {Vol} \bigl(Z^{+}_{h}(t)\bigr)d(t-t_{h})^{p} \biggr)^{\frac{1}{p}} \\ &= \biggl( p \int_{0}^{\infty}\operatorname{Vol}\bigl(Z^{+}_{h}(t_{h} +t)\bigr)t^{p-1}\,dt \biggr)^{\frac{1}{p}} \\ & = \biggl( p \int_{0}^{\infty}\operatorname{Vol}\bigl(Z^{+}_{h}(t_{h}+t) \bigr)^{\frac {1}{p}} \operatorname{Vol}\bigl(Z^{+}_{h}(t_{h}+t) \bigr)^{1-\frac{1}{p}}t^{p-1}\,dt \biggr)^{\frac{1}{p}}. \end{aligned} $$

As $\operatorname{Vol}(Z^{+}_{h}(t))$ is a monotonically decreasing function of *t*, we have

$$ t \operatorname{Vol}\bigl(Z^{+}_{h}(t_{h}+t) \bigr)^{\frac{1}{p}} \leq \int_{0}^{t} \operatorname{Vol}\bigl(Z^{+}_{h}(t_{h} +\tau)\bigr)^{\frac{1}{p}} \,d\tau, $$

and hence

17 $$ t^{p-1} \operatorname{Vol}\bigl(Z^{+}_{h}(t_{h}+t) \bigr)^{\frac{p-1}{p}} \leq \biggl( \int _{0}^{t} \operatorname{Vol} \bigl(Z^{+}_{h}(t_{h} + \tau)\bigr)^{\frac{1}{p}} \,d\tau \biggr)^{p-1}. $$

Using inequality (17) and by putting $g(t)=\int_{0}^{t} \operatorname{Vol}(Z^{+}_{h}(t_{h} + \tau))^{\frac{1}{p}} \,d \tau$, equation (16) becomes

$$ \biggl( \int_{\Gamma}\bigl(h(x)-t_{h} \bigr)_{+}^{p} \,dx \biggr)^{\frac{1}{p}} \leq \biggl( \int_{0}^{\infty}p g^{p-1}(t) g'(t) \,dt \biggr)^{\frac{1}{p}}. $$

This implies that

$$ \begin{aligned}[b] \biggl( \int_{\Gamma}\bigl(h(x)-t_{h} \bigr)_{+}^{p} \,dx \biggr)^{\frac{1}{p}} & \leq \int_{0}^{\infty}\operatorname{Vol}\bigl(Z^{+}_{h}(t_{h} +t)\bigr)^{\frac{1}{p}} \,dt \\ & = \int_{t_{h}}^{\infty}\operatorname{Vol}\bigl(Z^{+}_{h}(t) \bigr)^{\frac{1}{p}} \,dt, \end{aligned} $$

which is the desired inequality (14).

To prove the second inequality, we consider

18 $$ \begin{aligned}[b] \biggl( \int_{\Gamma}\bigl(h(x)-t_{h} \bigr)_{-}^{p} \,dx \biggr)^{\frac{1}{p}} & = \biggl( \int_{0}^{\infty}\operatorname{Vol}\bigl(Z^{+}_{h}(t) \bigr) d(t-t_{h})_{-}^{p} \biggr)^{\frac{1}{p}} \\ & = \biggl( \int_{0}^{t_{h}} \operatorname{Vol} \bigl(Z^{+}_{h}(t)\bigr)d(t_{h}-t)^{p} \biggr)^{\frac{1}{p}} \\ &= \biggl( p \int_{0}^{t_{h}} -\operatorname {Vol} \bigl(Z^{+}_{h}(t)\bigr) (t_{h}-t)^{p-1}\,dt \biggr)^{\frac{1}{p}}. \end{aligned} $$

As $-\operatorname{Vol}(Z^{+}_{h}(t))\leq-\operatorname{Vol}(Z^{-}_{h}(t))\leq \operatorname{Vol}(Z^{-}_{h}(t))$ for $t < t_{h}$, equation (18) becomes

19 $$ \biggl( \int_{\Gamma}\bigl(h(x)-t_{h} \bigr)_{-}^{p} \,dx \biggr)^{\frac{1}{p}} \leq \biggl( p \int_{0}^{t_{h}} \operatorname {Vol} \bigl(Z^{-}_{h}(t)\bigr) (t_{h}-t)^{p-1}\,dt \biggr)^{\frac{1}{p}}. $$

Now $\operatorname{Vol}(Z^{-}_{h}(t))$ is a monotonically increasing function of *t* and therefore

20 $$ (t_{h}-t )^{p-1} \operatorname{Vol} \bigl(Z^{-}_{h}(t)\bigr)^{\frac{p-1}{p}} \leq \biggl( \int_{t}^{t_{h}} \operatorname{Vol}\bigl(Z^{-}_{h}( \tau)\bigr)^{\frac{1}{p}} \,d\tau \biggr)^{p-1}. $$

Using inequality (20) and by putting $q(t)=\int_{t}^{t_{h}} \operatorname{Vol}(Z^{-}_{h}(\tau))^{\frac{1}{p}} \,d \tau$, inequality (19) becomes

$$ \biggl( \int_{\Gamma}\bigl(h(x)-t_{h} \bigr)_{-}^{p} \,dx \biggr)^{\frac{1}{p}} \leq \biggl( \int_{0}^{t_{h}} - p q^{p-1}(t) q'(t) \,dt \biggr)^{\frac{1}{p}}. $$

This implies that

$$ \biggl( \int_{\Gamma}\bigl(h(x)-t_{h} \bigr)_{-}^{p} \,dx \biggr)^{\frac{1}{p}} \leq \int_{0}^{t_{h}} \operatorname{Vol} \bigl(Z^{-}_{h}(t)\bigr)^{\frac{1}{p}} \,dt. $$

 □

### Proof of Theorem 2.1

Define the set $Z_{h}(t)$ as

$$ Z_{h}(t)= \textstyle\begin{cases} Z^{+}_{h}(t), & \mbox{if } t > t_{h},\\ Z^{-}_{h}(t), & \mbox{if } t\leq t_{h}. \end{cases} $$

We know that $\operatorname{Vol}(Z^{+}_{h}(t))\leq\operatorname {Vol}(Z^{-}_{h}(t))= \operatorname{Vol}(\bar{Z}^{+}_{h}(t))$ for all $t > t_{h}$ and $\operatorname{Vol}(Z^{-}_{h}(t))\leq [4]\operatorname {Vol}(Z^{+}_{h}(t))=\operatorname{Vol}(\bar{Z}^{-}_{h}(t))$ for all $t\leq t_{h}$. Therefore,

21 $$ \bigl\vert \partial Z^{+}_{h}(t) \bigr\vert \geq C_{\gamma}\bigl(\operatorname{Vol}\bigl(Z^{+}_{h}(t)\bigr) \bigr)^{\frac {{\gamma-1}}{{\gamma}}} \quad\mbox{if } t > t_{h} $$

and

22 $$ \bigl\vert \partial Z^{-}_{h}(t) \bigr\vert \geq C_{\gamma}\bigl(\operatorname{Vol}\bigl(Z^{-}_{h}(t)\bigr) \bigr)^{\frac {{\gamma-1}}{{\gamma}}} \quad\mbox{if } t \leq t_{h}. $$

In order to prove the theorem, we need the co-area formula

$$ \int_{\Gamma}\bigl\vert h'(x) \bigr\vert \,dx = \int_{0}^{\infty}\bigl\vert \partial Z_{h}(t) \bigr\vert \,dt, $$

or

23 $$ \int_{\Gamma}\bigl\vert h'(x) \bigr\vert \,dx = \int_{0}^{t_{h}} \bigl\vert \partial Z^{-}_{h}(t) \bigr\vert \,dt+ \int _{t_{h}}^{\infty}\bigl\vert \partial Z^{+}_{h}(t) \bigr\vert \,dt. $$

Equation (23), along with inequalities (21) and (22). implies that

$$ \int_{\Gamma}\bigl\vert h'(x) \bigr\vert \,dx \geq C_{\gamma}\biggl( \int_{0}^{t_{h}} \bigl(\operatorname {Vol} \bigl(Z^{-}_{h}(t)\bigr)\bigr)^{\frac{{\gamma-1}}{{\gamma}}} \,dt+ \int_{t_{h}}^{\infty}\bigl(\operatorname{Vol} \bigl(Z^{+}_{h}(t)\bigr)\bigr)^{\frac{{\gamma-1}}{{\gamma}}} \,dt \biggr), $$

which, due to Lemma 3.1, gives

$$ \begin{aligned}[b] \int_{\Gamma}\bigl\vert h'(x) \bigr\vert \,dx & \geq C_{\gamma}\biggl( \int_{\Gamma}\bigl(h(x)-t_{h}\bigr)_{-}^{\frac{\gamma}{\gamma-1}} \,dx \biggr)^{\frac{\gamma -1}{\gamma}}+C_{\gamma}\biggl( \int_{\Gamma}\bigl(h(x)-t_{h}\bigr)_{+}^{\frac{\gamma }{\gamma-1}} \biggr)^{\frac{\gamma-1}{\gamma}} \\ & = C_{\gamma}\biggl( \int_{\Gamma}\bigl\vert h(x)-t_{h} \bigr\vert ^{\frac{\gamma}{\gamma-1}} \,dx \biggr)^{\frac{\gamma-1}{\gamma}}. \end{aligned} $$

Here we used $|h(x)-t_{h}|^{p}=(h(x)-t_{h})_{+}^{p}+(h(x)-t_{h})_{-}^{p}$.

### Proof of Corollary 2.2

Inequality (11) in particular implies that

24 $$ \int_{\Gamma}\bigl\vert h'(x) \bigr\vert \,dx \geq C_{\gamma}\biggl( \int_{\Gamma}\bigl(h(x)-t_{h}\bigr)_{+}^{\frac{\gamma}{\gamma-1}} \,dx \biggr)^{\frac{\gamma -1}{\gamma}}. $$

For $\beta\geq1$, $a>0$ and $b>0$, the following elementary inequality holds:

25 $$ (a+b)^{\beta}\leq2^{\beta-1}\bigl(a^{\beta}+b^{\beta}\bigr). $$

If we choose $a=(h-t_{h})_{+}$, $b=t_{h}$ and use the fact that $(h-t_{h})_{+}\geq h$, inequality (25) yields

$$(h-t_{h})_{+}^{\beta}\geq2^{1-\beta} h^{\beta}-t_{h}^{\beta}. $$

Therefore,

26 $$ \begin{aligned}[b] \int_{\Gamma}\bigl(h(x)-t_{h}\bigr)_{+}^{\beta}\,dx &= \int_{Z^{+}_{h}(0)}\bigl(h(x)-t_{h}\bigr)_{+}^{\beta}\,dx \\ & \geq 2^{1-\beta} \int_{Z^{+}_{h}(0)} h^{\beta}(x) \,dx- t_{h}^{\beta}\operatorname{Vol}\bigl(Z^{+}_{h}(0)\bigr) \\ &\geq \biggl[2^{1-\beta} \int_{Z^{+}_{h}(0)} h^{\beta}(x) \,dx- t_{h}^{\beta}\operatorname{Vol}\bigl(Z^{+}_{h}(0)\bigr) \biggr]_{+}. \end{aligned} $$

By another elementary inequality

$$(a-b)_{+}^{1/\beta}\geq a^{1/\beta}-b^{1/\beta}, $$

we obtain

27 $$\begin{aligned} \biggl[2^{1-\beta} \int_{Z^{+}_{h}(0)} h^{\beta}(x) \,dx- t_{h}^{\beta}\operatorname{Vol}\bigl(Z^{+}_{h}(0)\bigr) \biggr]_{+}^{\frac{1}{\beta}} & \geq2^{\frac {1}{\beta}-1} \biggl[ \int_{Z^{+}_{h}(0)} h^{\beta}(x) \,dx \biggr]^{\frac{1}{ \beta }} \\ & \quad{}-t_{h} \operatorname{Vol}\bigl(Z^{+}_{h}(0) \bigr)^{\frac{1}{\beta}}. \end{aligned}$$

Finally, choosing $\beta=\frac{\gamma}{\gamma-1}$ and combining inequalities (24), (26) and (27), we obtain the desired inequality (12).

### Proof of Theorem 2.3

We apply estimate (12) to $h^{q}$ assuming $h\geq0$, $h \in C^{1}(\Gamma)$ and $q>1$ is an integer, which gives

$$ \biggl( \int_{\Gamma}\bigl\vert h^{q}(x) \bigr\vert ^{\frac{\gamma}{\gamma-1}} \,dx \biggr)^{\frac {\gamma-1}{\gamma}}- 2^{\frac{1}{\gamma}}t_{h^{q}} \bigl(\operatorname {Vol}\bigl(Z^{+}_{h^{q}}(0)\bigr) \bigr)^{\frac{\gamma-1}{\gamma}} \leq\frac{2^{\frac {1}{\gamma}}}{C_{\gamma}} \int_{\Gamma}\bigl\vert \bigl(h^{q} \bigr)'(x) \bigr\vert \,dx. $$

Putting $\Theta(h,\gamma,q,\Gamma)=2^{1/\gamma} t_{h^{q}}\operatorname {Vol}(Z^{+}_{h^{q}}(0))^{\frac{\gamma-1}{\gamma}}$, the above inequality becomes

$$\begin{aligned}& \biggl( \int_{\Gamma}\bigl\vert h^{q}(x) \bigr\vert ^{\frac{\gamma}{\gamma-1}} \,dx \biggr)^{\frac {\gamma-1}{\gamma}}-\Theta(h,\gamma,q,\Gamma) \leq \frac{2^{\frac {1}{\gamma}}}{C_{\gamma}} \int_{\Gamma}\bigl\vert \bigl(h^{q} \bigr)'(x) \bigr\vert \,dx, \\& \begin{aligned} &\biggl( \int_{\Gamma}\bigl\vert h(x) \bigr\vert ^{\frac{q\gamma}{\gamma-1}} \,dx \biggr)^{\frac {\gamma-1}{\gamma}}-\Theta(h,\gamma,q,\Gamma)\\ &\quad \leq \frac{q 2^{\frac {1}{\gamma}}}{C_{\gamma}} \int_{\Gamma}\bigl\vert \bigl(h^{q-1}\bigr) (x) \bigr\vert \bigl\vert h'(x) \bigr\vert \,dx \\ &\quad \leq \frac{q 2^{\frac{1}{\gamma}}}{C_{\gamma}} \biggl( \int_{\Gamma}\bigl\vert h(x) \bigr\vert ^{(q-1)\frac{p}{p-1}} \,dx \biggr)^{\frac{p-1}{p}} \biggl( \int_{\Gamma}\bigl\vert h'(x) \bigr\vert ^{p} \,dx \biggr)^{\frac{1}{p}}. \end{aligned} \end{aligned}$$

We choose *p* such that $\frac{q\gamma}{\gamma-1} = \frac {p(q-1)}{p-1}$, that is,

$$p = \frac{q \gamma}{q+ \gamma-1}. $$

With this choice of *p*, the above inequality becomes

$$\begin{aligned} \biggl( \int_{\Gamma}\bigl\vert h'(x) \bigr\vert ^{\frac{q \gamma}{q + \gamma-1}} \,dx \biggr)^{\frac{q+\gamma-1}{q \gamma}} & \geq C_{\gamma,q} \biggl( \int_{\Gamma}\bigl\vert h(x) \bigr\vert ^{\frac{q \gamma}{\gamma-1}} \,dx \biggr)^{\frac{\gamma-1}{q\gamma }} \\ & \quad{}- C_{\gamma,q}\Theta(h,\gamma,q,\Gamma) \biggl( \int_{\Gamma}\bigl\vert h(x) \bigr\vert ^{\frac{q \gamma}{\gamma-1}} \,dx \biggr)^{\frac{(\gamma -1)(1-q)}{q\gamma}}, \end{aligned}$$

where $C_{\gamma,q}=\frac{2^{-1/\gamma}C_{\gamma}}{q}$.

## Sobolev inequalities involving Cheeger constant

Finding an optimal cut of a graph into two or more disjoint subsets is one of the fundamental problems in graph theory. Cheeger cut, which we define below, among several kinds of balanced graph cut, is the most widely used tool to obtain optimal partitioning of a graph. The *Cheeger constant* $C_{\Gamma}$ of a metric graph Γ is defined as

$$C_{\Gamma}:= \inf\frac{ \vert \partial Z \vert }{\min\{\operatorname{Vol}(Z), \operatorname{Vol}(\bar{Z})\}} $$

where inf is taken over all Lebesgue measurable open subsets *Z* of the metric graph. The partition $(Z, \bar{Z})$ of Γ is called a *Cheeger cut* if

$$\frac{ \vert \partial Z \vert }{\min\{\operatorname{Vol}(Z), \operatorname {Vol}(\bar{Z})\}} = C_{\Gamma}. $$

In order to find the Cheeger cut of a metric graph, one would really need to know the value of the associated Cheeger constant or at least an approximation of it. The most well-known technique to approximate the Cheeger constant is via lowest non-zero eigenvalue of the standard Laplace operator. Precisely, if $\lambda_{1}$ denotes the lowest non-zero eigenvalue of the Laplace operator (6) subject to standard conditions (7) at the vertices, then the Cheeger inequality

$$\lambda_{1} \geq\frac{C_{\Gamma}^{2}}{4} $$

provides an upper bound on the Cheeger constant. For more details on the theory of Cheeger constants for quantum graph, we refer to the review article [17].

It is easy to see that $C_{\Gamma}=\lim_{\gamma\rightarrow\infty} C_{\gamma}$, where $C_{\gamma}$ is the isoperimetric constant of the graph defined in (8). Using this observation, one can rewrite the inequalities obtained in Theorems 2.1 and 2.3 in terms of the graph Cheeger constant. The obtained inequalities in the following theorems could be used as an alternative to estimate the Cheeger constant.

### Theorem 4.1

*Let* $C_{\Gamma}$ *be the Cheeger constant of the graph* Γ. *Then*, *for a non*-*negative function* $h \in C^{1}(\Gamma)$, *the following inequality holds*:

28 $$ \int_{\Gamma}\bigl\vert h'(x) \bigr\vert \,dx \geq C_{\Gamma}\int_{\Gamma}\bigl\vert h(x)-t_{h} \bigr\vert \,dx. $$

### Corollary 4.2

*Let* *h* *satisfy the conditions of the above theorem*. *Then the following inequality holds*:

29 $$ \int_{\Gamma}\bigl\vert h'(x) \bigr\vert \,dx \geq C_{\Gamma}\int_{\Gamma}\bigl\vert h(x) \bigr\vert \,dx -C_{\Gamma}t_{h} \operatorname{Vol}\bigl(Z^{+}_{h}(0)\bigr), $$

*where* $Z^{+}_{h}(0)=\{x\in\Gamma: h(x)>0\}$.

### Theorem 4.3

*For* $h \in C^{1}(\Gamma)$, $h \geq0$ *and for integer* $q\geq2$, *the following inequality holds*:

30 $$ \biggl( \int_{\Gamma}\bigl\vert h'(x) \bigr\vert ^{q} \,dx \biggr)^{\frac{1}{q}} \geq\frac{C_{\Gamma}}{q} \biggl[ \biggl( \int_{\Gamma}\bigl\vert h(x) \bigr\vert ^{q} \,dx \biggr)^{\frac{1}{q}}- \widetilde{\Theta}(h, q,\Gamma) \biggl( \int_{\Gamma}\bigl\vert h(x) \bigr\vert ^{q} \,dx \biggr)^{\frac{(1-q)}{q}} \biggr], $$

*where* $\widetilde{\Theta}(h,q,\Gamma)= t_{h^{q}}\operatorname{Vol}(Z^{+}_{h^{q}}(0))$.

The following result follows from Theorem 4.3 and is commonly known as Cheeger inequality. It was first proved by Nicaise [25, Theorem 3.2].

### Corollary 4.4

*Let* $\lambda_{1}$ *be the lowest non*-*zero eigenvalue of the Laplace operator* *H* *given by* (6) *with the standard or Kirchhoff vertex conditions* (7) *defined in* $L_{2}(\Gamma)$, *where* Γ *is a compact and connected metric graph*. *Then we have*

31 $$ \lambda_{1} \geq\frac{C_{\Gamma}^{2}}{4}. $$

### Proof

The lowest eigenvalue of the operator *H* is zero and corresponding eigenfunction is a constant. Let $\psi_{1}$ denote the eigenfunction corresponding to the lowest non-zero eigenvalue $\lambda_{1}$. We can assume $\psi_{1}$ to be real-valued. We define

$$\Gamma_{+}:=\bigl\{ x\in\Gamma \mid \psi_{1}(x)>0\bigr\} $$

and

$${\tilde{\psi }}_{1}:=1_{\Gamma_{+}}\psi_{1}.$$

Clearly, $\widetilde{\psi}_{1}$ disappears on the boundary of $\Gamma_{+}$. By min–max principle, we have

$$\lambda_{1}=\frac{\langle-\widetilde{\psi}_{1}'', \widetilde{\psi}_{1}\rangle }{\langle\widetilde{\psi}_{1}, \widetilde{\psi}_{1}\rangle}=\frac{\int _{\Gamma} \vert \widetilde{\psi}'_{1}(x) \vert ^{2} \,dx}{\int_{\Gamma} \vert \widetilde{\psi }_{1}(x) \vert ^{2} \,dx}\geq \frac{C_{\Gamma}^{2}}{4}. $$

Here we used integration by parts and applied inequality (30) to $\widetilde{\psi}_{1}$ with $q=2$ along with the fact that the mean value of the eigenfunction is 0. Note that $\widetilde {\Theta}(\widetilde{\psi}_{1},2,\Gamma)=0$. □

## Conclusions

Metric graphs are locally one-dimensional objects. We first defined isoperimetric dimension of these graphs which may be greater than one and may not be an integer. We obtained Sobolev type inequalities for such graphs. These inequalities depend on the isoperimetric dimension of the graph. Moreover, we gave versions of these inequalities that involve graph’s Cheeger constant. Functional inequalities are important tools for the study of spectral properties of differential operators. We demonstrated this connection by obtaining a previously known lower bound on the first non-zero eigenvalue of the Laplace operator defined on a metric graph.

## References

[1] Adami R., Serra E., Tilli P. (2017). Negative energy ground states for the $L^{2}$-critical NLSE on metric graphs. Commun. Math. Phys..

[2] Baker M., Faber X.W.C. (2006). Metrized graphs, Laplacian operators, and electrical networks. Quantum Graphs and Their Applications.

[3] Berkolaiko G., Kuchment P. (2013). Introduction to Quantum Graphs.

[4] Brüning J., Geyler V., Pankrashkin K. (2008). Spectra of self-adjoint extensions and applications to solvable Schrödinger operators. Rev. Math. Phys..

[5] Cartwright D.I., Woess W. (2007). The spectrum of the averaging operator on a network (metric graph). Ill. J. Math..

[6] Cattaneo C. (1997). The spectrum of the continuous Laplacian on a graph. Monatshefte Math..

[7] Cheeger J. (1970). A lower bound for the smallest eigenvalue of the Laplacian. Problems in Analysis: A Symposium in Honor of Salomon Bochner.

[8] Chung F.R.K. (1997). Spectral Graph Theory.

[9] Chung F.R.K., Yau S.T. (1995). Eigenvalues of graphs and Sobolev inequalities. Comb. Probab. Comput..

[10] Davies E.B. (1989). Heat Kernels and Spectral Theory.

[11] Evans L.C. (1998). Partial Differential Equations.

[12] Exner P., Šeba P. (1989). Free quantum motion on a branching graph. Rep. Math. Phys..

[13] Figotin A., Kuchment P. (1998). Spectral properties of classical waves in high contrast periodic media. SIAM J. Appl. Math..

[14] Gagliardo E. (1958). Proprietà di alcune classi di funzioni in più variabili. Ric. Mat..

[15] Gnutzmann S., Smilansky U. (2006). Quantum graphs: applications to quantum chaos and universal spectral statistics. Adv. Phys..

[16] Harmer M. (2000). Hermitian symplectic geometry and extension theory. J. Phys. A, Math. Gen..

[17] Kennedy J.B., Mugnolo D. (2016). The Cheeger constant of a quantum graph. Proc. Appl. Math. Mech..

[18] Kostrykin V., Schrader R. (1999). Kirchhoff’s rule for quantum wires. J. Phys. A, Math. Gen..

[19] Kottos T., Smilansky U. (1997). Quantum chaos on graphs. Phys. Rev. Lett..

[20] Kuchment P. (2004). Quantum graphs: I. Some basic structures. Waves Random Media.

[21] Kuchment P. (2005). Quantum graphs: II. Some spectral properties of quantum and combinatorial graphs. J. Phys. A, Math. Gen..

[22] Kurasov, P.: Quantum graphs: spectral theory and inverse problems. In press

[23] Maz’ya V.G. (1985). Sobolev Spaces.

[24] Maz’ya V.G. (2003). Lectures on isoperimetric and isocapacitary inequalities in the theory of Sobolev spaces. Contemp. Math..

[25] Nicaise S. (1987). Spectre des réseaux topologiques finis. Bull. Sci. Math. (2).

[26] Nirenberg L. (1959). On elliptic partial differential equations. Ann. Sc. Norm. Super. Pisa.

[27] Pankrashkin K. (2006). Spectra of Schrödinger operators on equilateral quantum graphs. Lett. Math. Phys..

[28] Post O. (2009). Spectral analysis of metric graphs and related spaces. Limits of Graphs in Group Theory and Computer Science (Bernoulli Center, Lausanne).

[29] Post O. (2012). Spectral Analysis on Graph-Like Spaces.

[30] Sobolev S. (1963). On a theorem of functional analysis. Transl. Am. Math. Soc.ger.

[31] von Below J. (1985). A characteristic equation associated to an eigenvalue problem on $C^{2}$-networks. Linear Algebra Appl..

